# Lipo-Chitooligosaccharides (LCOs) as Elicitors of the Enzymatic Activities Related to ROS Scavenging to Alleviate Oxidative Stress Generated in Tomato Plants under Stress by UV-B Radiation

**DOI:** 10.3390/plants11091246

**Published:** 2022-05-05

**Authors:** José A. Lucas, Ana García-Villaraco, Beatriz Ramos-Solano, Khalid Akdi, Francisco Javier Gutierrez-Mañero

**Affiliations:** 1 Plant Physiology, Pharmaceutical and Health Sciences Department, Faculty of Pharmacy, Universidad San Pablo-CEU Universities, 28668 Boadilla del Monte, Spain; anabec.fcex@ceu.es (A.G.-V.); bramsol@ceu.es (B.R.-S.); jgutierrez.fcex@ceu.es (F.J.G.-M.); 2 Trichodex S.A., Polígono Industrial La Isla, Rio Viejo 57-59, 41703 Sevilla, Spain; khalid.akdi@trichodex.com

**Keywords:** antioxidant defense enzymes, flavonoids, lipo-chitooligosaccharides (LCOs), MDA, oxidative stress, ROS, tomato, ultraviolet-B radiation

## Abstract

Exposure to ultraviolet-B (UV-B) radiation can lead to oxidative damage in plants, increasing reactive oxygen species (ROS) production. To overcome ROS burst, plants have antioxidant mechanisms related to ROS scavenging which can be improved by elicitation with biological agents or derived molecules (elicitors), as they can trigger a physiological alert state called “priming”. This work describes the effects of lipo-chitooligosaccharides (LCOs) treatment applied to tomato plants under UV-B stress. The LCOs used in the study are produced by three species of the genus Ensifer (formerly *Sinorhizobium*) (SinCEU-1, SinCEU-2, and SinCEU-3) were assayed on tomato plants under UV-B stress. LCOs were able to significantly increase most of the enzymatic activities related to ROS scavenging while non-enzymatic antioxidants were not modified. This response was associated with a lower oxidative stress, according to malondialdehyde (MDA) levels and the higher antioxidant capacity of the plants. Furthermore, the photosynthetic efficiency of LCOs-treated plants indicated a better physiological state than the control plants. Therefore, although more studies and deepening of certain aspects are necessary, LCOs have shown great potential to protect plants from high UV-B radiation conditions.

## 1. Introduction

Plants are exposed to different stress factors that threaten or hinder growth and development, causing losses in crop productivity and quality that account for multi-billion-dollar losses in yield (FAO 2017 Report, http://www.fao.org/3/I8656EN/i8656en.pdf (accessed on 6 January 2022)). Stress situations include both biotic factors such as fungal pathogens and insects, as well abiotic factors such as drought, salinity, and heat, among others [1,2].

One of the components of solar radiation that naturally reaches the earth is ultraviolet (UV) radiation. This radiation can be classified into three types depending on the wavelength: UV-A (315–390 nm), UV-B (280–315 nm), and UV-C (100–280 nm). The amount of ultraviolet radiation reaching the earth’s surface depends on the thickness of the ozone layer, which is rapidly being depleted by the alarming emission of anthropogenic air pollutants such as nitrogen oxides and chlorofluorocarbons [3,4,5]. UV-B radiation represents <0.5% of the total solar energy that reaches the earth’s surface, but this percentage increases due to the degradation of the ozone layer [6,7].

It is well known that UV-B can damage photosynthetic machinery processes, proteins, and DNA and arrests the cell cycle [8,9]. UV-B radiation is a major source of ROS, like O_2_^−^ and H_2_O_2_ [10], which in turn cause biomolecules oxidation [11,12,13], because they are extremely cytotoxic and reactive [14].

Plants are genetically endowed with enzymatic and non-enzymatic antioxidants to overcome ROS bursts [10]. The main enzymatic antioxidants include superoxide dismutase (SOD), catalase (CAT), peroxidase (POX), and all enzymes involved in the ascorbate–glutathione cycle [15,16]. Non-enzymatic antioxidant molecules include ascorbate (ASA), glutathione (GSH), jasmonic acid (JA), polyphenols, carotenoids, etc. [17]. Polyphenols are among the largest and most complex family of antioxidants. Some polyphenols, such as flavonoids (e.g., quercetin and luteolin) and hydroxycinnamic acids (e.g., caffeic, ferulic, and sinapic acids) mainly accumulate in the leaf epidermal cells, blocking UV-B radiation from reaching photosynthetic leaf tissues [18,19]. In addition, polyphenols participate as antioxidants, scavenging ROS, such as O_2_^−^, OH^•^ and ^1^O_2_ [19]. Upon stress, ROS production may dramatically increase, and plant scavenging systems cannot cope with this increase, resulting in oxidative damage [20,21,22].

The chloroplasts, peroxisome, mitochondria, cell wall, and plasma membrane are the main cell compartments where ROS are produced. In stress conditions, ROS levels increase, mainly through a reduction in electron transport in the Calvin cycle and a higher electron leakage during photosynthesis in the Mehler reaction. Together, this results in higher respiration and lower photosynthesis and elevated ROS levels in stressed tissues [23]. ROS participates in stress signaling through the transduction of signals from mitogen-activated protein kinases (MAPKs) [24], which leads to the induction of several pathways and activation of gene expression related to antioxidant systems with the objective of adjusting mainly the levels of H_2_O_2_ [25], since H_2_O_2_ is the most stable and easily disseminated form of oxidative stress.

In addition, and interconnected with different cascades of MAPKs, the genes that express for the different enzymatic activities of the ascorbate—glutathione cycle have been shown to be up-regulated in stress situations. There are other genes, such as glutathione-S-transferases (GST), not involved in the ascorbate—glutathione cycle, which have been shown to increase their expression in stressful situations, enhancing tolerance to oxidative damage [26].

In view of the dramatic consequences of uncontrolled oxidative stress, plants still use additional help such as that provided by beneficial microorganisms, known as plant growth-promoting bacteria (PGPB). These bacteria and some of their elicitors (structural molecules or metabolic molecules released to the medium) can induce a physiological alert state prior to stress challenge known as priming [27]; primed plants are able to develop a faster and/or stronger activation of different mechanisms in response to biotic or abiotic stress [28,29,30,31,32].

Among the best-studied bacterial elicitors are lipo-chitooligosaccharides (LCOs) or nodulation factors (Nod factors). LCOs are compounds produced by rhizobia during the establishment of their symbiotic relationship with legumes. LCOs are composed by two-six β-1,4-linked *N*-acetyl-d-glucosamine (GlcNAc) residues, and a fatty acid group attached to the N-terminal residue of the non-reducing end. Fatty acid structure varies from species to species [33]. At very low concentrations, LCOs induce responses in the host plant, such as root hair deformation, calcium spiking, membrane depolarization and formation of pre-infection threads and nodule primordia initiation [33,34,35].

Interestingly, Nod factors may also induce responses in non-legume plants, i.e., acceleration of flowering and fruiting in tomato [36], with the corresponding improvement of tomato fruit yield; or stimulating photosynthesis and increasing leaf area and dry matter in corn [37]. LCOs have also been used to improve the physiological state of plants subjected to saline stress [38,39]. However, this is the first study on the influence of the LCOs treatment on antioxidant status in a plant exposed to UV-B stress, not involving the elucidation of the mechanisms of action of LCOs at the level of gene expression, which will have to be thoroughly studied once the ability of these molecules to elicit scavenging mechanisms of ROS has been demonstrated in this stressful situation.

Therefore, the objective of this study was to determine the ability of LCOs from three strains of *Ensifer* spp. (formerly *Sinorhizobium***;** two strains belonging to the species *Ensifer fredii* and one to *Ensifer arboris*), to improve adaptation of tomato plants to UV-B radiation stress, evaluating their capacity to trigger antioxidant systems, improving ROS scavenging capacity upon stress as compared to control plants. To achieve this goal, enzymatic activities related to ROS scavenging and polyphenol concentration were studied to assess the antioxidant capacity of the plants. In addition, photosynthetic efficiency and malondialdehyde (MDA) as markers of physiological stress and oxidative stress, were measured respectively.

## 2. Results

First, lipo-chitooligosaccharides from the three bacterial strains were isolated, purified and their biological activity was determined using an in vitro root hair deformation assay in soybean [40]. Then, the ability of LCOs to stimulate plant growth and flowering was evaluated in tomato plants to select the most effective concentration in order to finally evaluate the ability to improve adaptation to UV-B radiation stress.

In the presence of naringenin and in the conditions indicated in Materials and Methods, strains were able to produce LCOs in the following concentrations: SinCEU-1, 2.20 ng mL^−1^; SinCEU-2, 4.42 ng mL^−1^; SinCEU-3, 2.76 ng mL^−1^.

The percentage of root hair deformation produced by LCOs from SinCEU-1 and SinCEU-2 was only significant with a concentration of 10^−8^ M, while effects of LCOs from SinCEU-3 were significant at all concentrations used (Table 1).

### 2.1. Effect of LCOs on Height and Flowering of Tomato Plants

**Figure 1:**
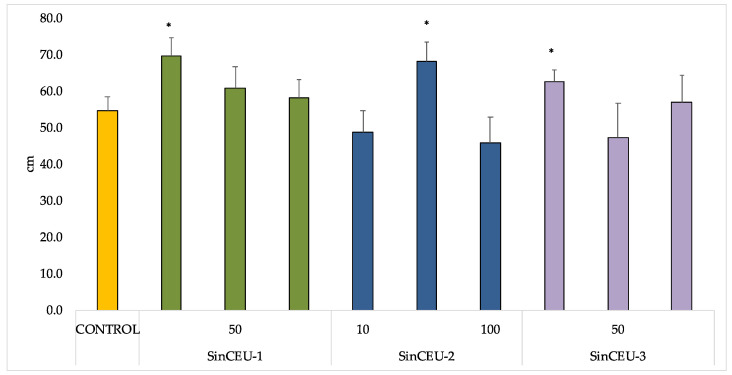
Height of tomato plants (cm) treated with LCOs from SinCEU-1, SinCEU-2, and Sin CEU-3 at 10, 50, and 100 ng/plant. Plants were treated by foliar spray twice (1 week [first dose] and 1 month [second dose] after transplant); height was measured 1 week after the second treatment. Asterisks indicate significant differences with control (*p* < 0.05, *t*-test).

After demonstrating the biological activity of LCOs in vitro, an experiment on tomato plants was designed to evaluate the effects of LCOs on growth and flowering according to Chen et al. [33]. Only LCOs from SinCEU-1 at 10 ng (SinCEU-1 (10)), SinCEU-2 at 50 ng (SinCEU-2 (50)), and SinCEU-3 3 at 10 ng (SinCEU-3 (10)) significantly increased height (Figure 1).

**Figure 2:**
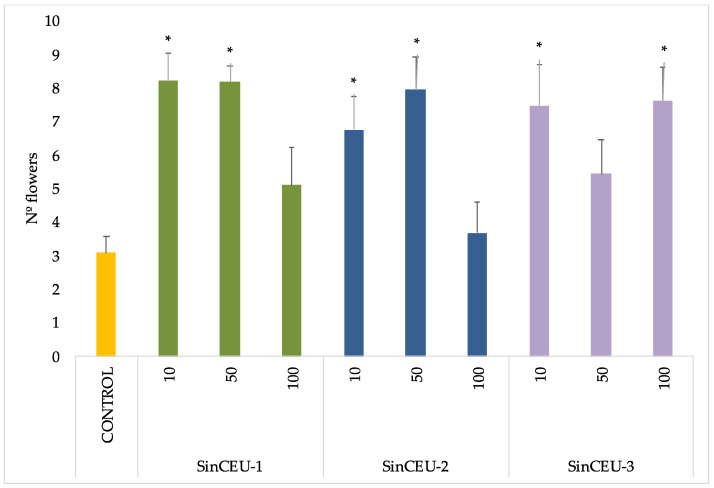
Number of flowers of tomato plants treated with LCOs from SinCEU-1, SinCEU-2, and Sin CEU-3 at 10, 50, and 100 ng/plants. This parameter was assessed 20 days after the second dose of LCOs. Asterisks indicate significant differences relative to control (*p* < 0.05, *t*-test).

The number of flowers was counted 20 days after the second dose, at fruit onset (Figure 2). All treatments except SinCEU-1 at 100 ng plant-1, SinCEU-2 at 100 ng plant-1, and SinCEU-3 at 50 ng plant-1 significantly increased the number of flowers.

### 2.2. Effects of LCOs on Tomato Plants Treated with UV-B Radiation

Based on results described above, LCOs from SinCEU-1 (10), SinCEU-2 (50), and SinCEU-3 (10) were used in an experiment on plants treated with UV-B radiation as described in Section 4.7.

### 2.3. Non-Enzymatic Antioxidants (Total Phenols and Total Flavonoids)

**Figure 3:**
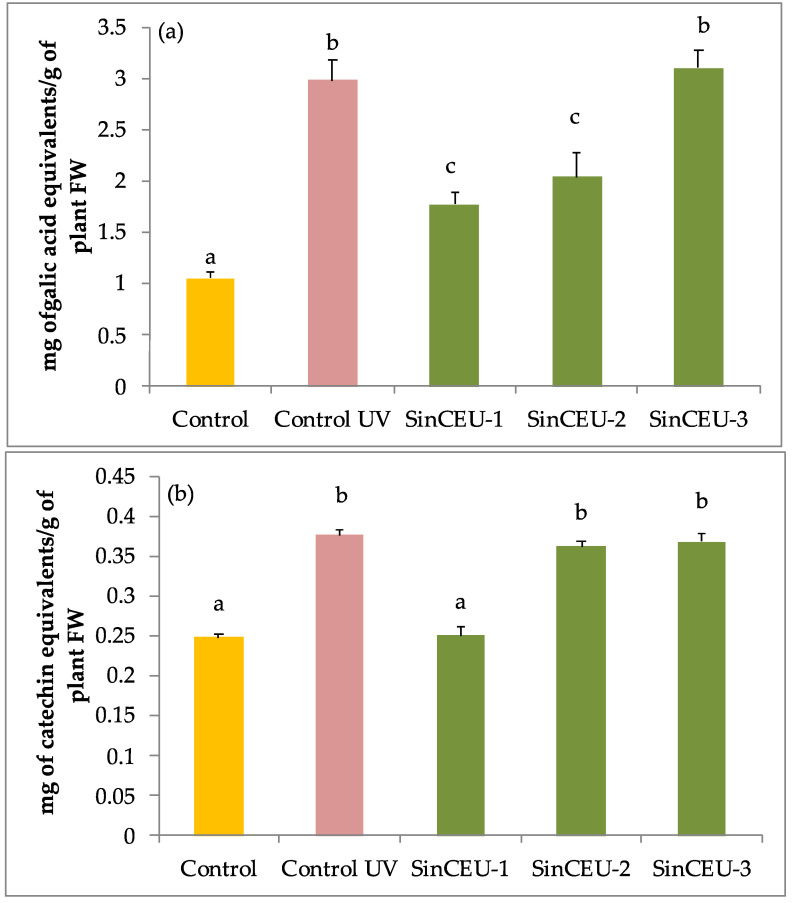
Total phenols and total flavonoids in tomato plants treated with LCOs from SinCEU-1 (10 ng/plant); SinCEU-2 (50 ng/plant); and SinCEU-3 (10 ng/plant) and irradiated with UV-B. (**a**) Total phenols expressed as mg of gallic acid equivalents per g FW; (**b**) total flavonoids content expressed as mg catechin equivalents per g FW. Letters indicate the statistical significance between treatments according to the LSD test (*p* < 0.05).

UV-B control plants showed a significant three-fold increase in total phenols (Figure 3a) and 30% increase in flavonols (Figure 3b) as compared to controls. As regards to phenols, SinCEU-3 (10) reached similar values to UV-controls, while SinCEU-1 (10) and SinCEU-2 (50) showed only a significant two-fold increase compared to non-stressed controls. In the case of flavonoids, SinCEU-1 (10) maintained levels of non-UV controls while SinCEU-2 (50) and SinCEU-3 (10) matched the higher values in UV-controls.

### 2.4. Enzyme Activities Related to Oxidative Stress

**Figure 4:**
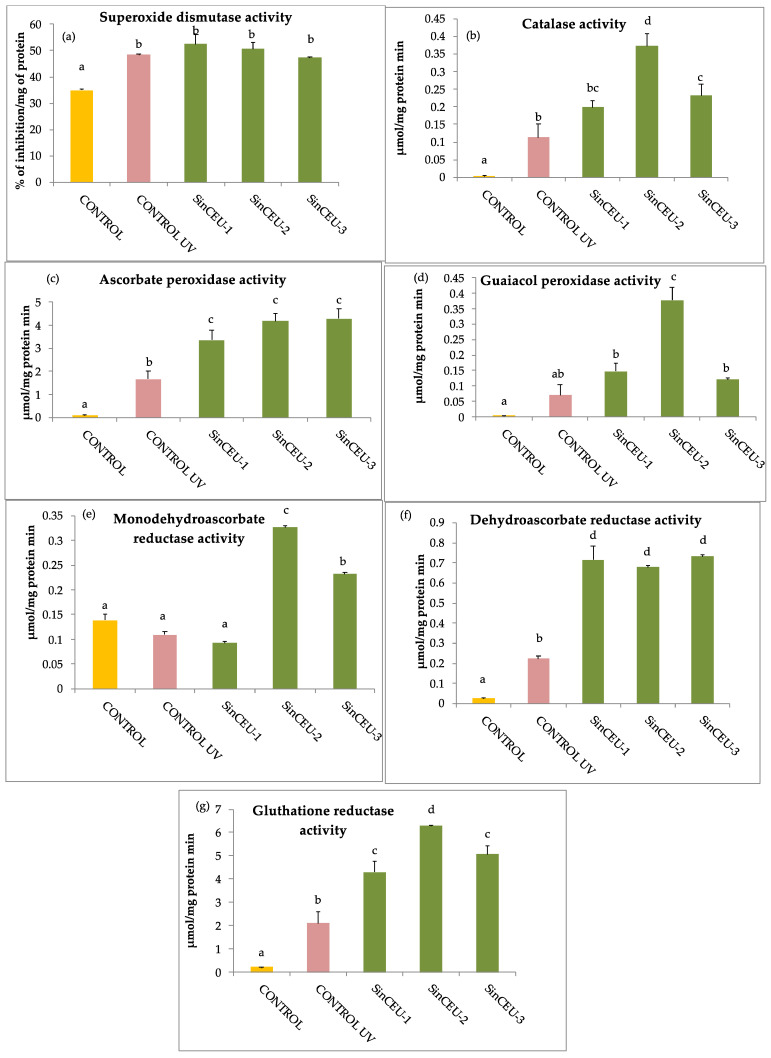
Enzyme activities related with ROS scavenging in tomato plants treated with LCOs from SinCEU-1 (10 ng/plant); SinCEU-2 (50 ng/plant); and SinCEU-3 (10 ng/plant) and irradiated with UV-B: (**a**) superoxide dismutase (SOD); (**b**) catalase (CAT); (**c**) ascorbate peroxidase (APX); (**d**) guaiacol peroxidase (GPX); (**e**) monodehydroascorbate reductase (MDHAR); (**f**) dehydroascorbate reductase (DHAR) and (**g**) glutathione reductase (GR). SOD is expressed as % inhibition mg protein^−1^, all other enzymes are expressed as μmol mg protein^−1^ min^−1^. Letters indicate the statistical significance between treatments and controls according to the LSD test (*p* < 0.05).

UV-B increased all enzymatic activities related to oxidative stress, as values were higher in the UV-B control plants than in non-stressed controls, except for MDHAR activity. SOD activity was not modified by any LCOs treatment with regard to UV-controls. However, LCOs treatments increased all other enzymatic activities with respect to UV-controls, except SinCEU-1 (10) for MDHAR activity. SinCEU-2 (50) stands out, with the highest values for CAT, GPX, MDHAR, and GR activities (Figure 4).

### 2.5. Antioxidant Activity

**Figure 5:**
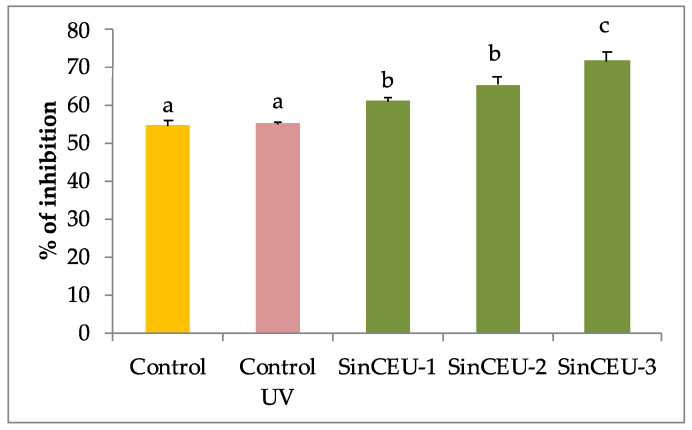
Antioxidant activity measured with β-carotene method in tomato plants treated with LCOs from SinCEU-1 (10 ng/plant), SinCEU-2 (50 ng/plant), and SinCEU-3 (10 ng/plant) and irradiated with UV-B. Letters indicate the statistical significance between treatments and controls according to the LSD test (*p* < 0.05).

UV-B did not increase antioxidant potential. However, all LCOs treatments significantly increased antioxidant activity with being SinCEU-3 (10) the most efficient (Figure 5).

### 2.6. Malondialdehyde (MDA)

**Figure 6:**
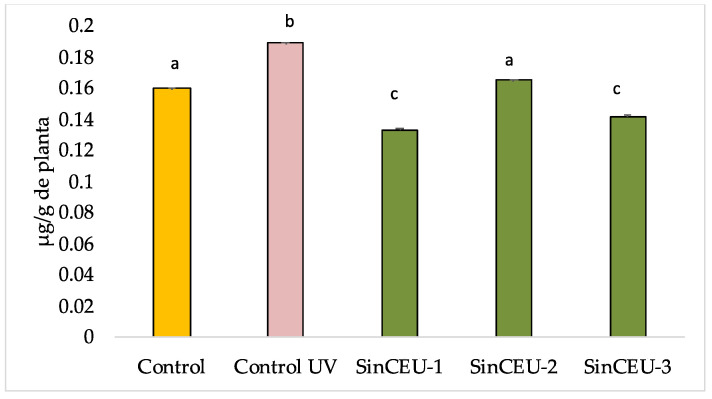
Concentration of Malondialdehyde (MDA) in tomato plants treated with LCOs from SinCEU-1 (10 ng/plant), SinCEU-2 (50 ng/plant), and SinCEU-3 (10 ng/plant) and irradiated with UV-B. MDA was measured as μg/g plant (FW). Different letters indicate significant differences (*p* < 0.05) according to LSD test.

The concentration of the oxidative stress marker (malondialdehyde; MDA) significantly increased in UV-controls. LCOs-treated plants showed significantly lower values compared to UV-B control (Figure 6). Interestingly, SinCEU-1 (10) and SinCEU-3 (10) were able to significantly reduce values below the non-stressed control.

### 2.7. Chlorophyll Fluorescence. Photosynthetic Efficiency

**Figure 7:**
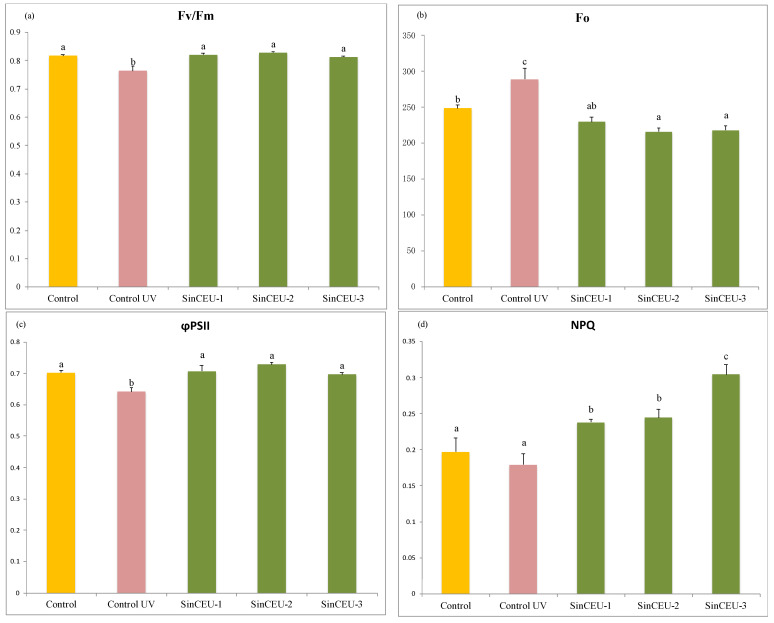
(**a**) Maximum photosynthetic efficiency of photosystem II (Fv/Fm), (**b**) fluorescence emission from dark adapted leaves (Fo), (**c**) effective quantum yield (φPSII) and (**d**) non-photochemical quenching (NPQ) in tomato plants treated with LCOs from SinCEU-1 (10 ng/plant), SinCEU-2 (50 ng/plant), and SinCEU-3 (10 ng/plant) and irradiated with UV-B. Different letters indicate significant differences (*p* < 0.05) according to LSD test.

Photosynthetic efficiency measured through the chlorophyll fluorescence emitted by photosystem II is shown in Figure 7 The maximum photosynthetic efficiency of photosystem II (Fv/Fm; Figure 7a), effective quantum yield (φPSII; Figure 7c), and non-photochemical quenching (NPQ; Figure 7d) significantly decreased in UV-controls, but values in LCOs-treated plants were significantly higher than UV-controls, maintaining values from non-stressed controls. Minimal fluorescence (Fo; Figure 7b) increased in UV-controls as compared to non-stressed controls, and LCOs-treated plants showed significantly lower values than UV-controls; in the case of SinCEU-2 (50) and SinCEU-3 (10), values were significantly lower than the non-stressed control.

## 3. Discussion

This work shows for the first time in the literature the ability of LCOs to alleviate oxidative stress associated to UV-B radiation in plants, focusing specifically on the study of ROS scavenging mechanisms, since the stress produced by UV-B radiation causes, among other things, the production of ROS [17].

LCOs, also called Nod factors, are signal molecules produced by rhizobia key for establishing symbiosis with legume plants [41,42]. More recently, the involvement of LCOs in the relationship between fungi and plants to establish mycorrhizae has also been described [43]. In addition, LCOs can stimulate several physiological responses in non-host plants including cell division [44], germination, seedling emergence and plant growth and yield in different plants [45], through processes other than nodulation [46]. They have also been used to improve the physiological state of plants subjected to saline stress [38,39].

The effectiveness of LCOs from 3 different rhizobia to modulate responses in legume plants, as well as its optimal concentration, have been demonstrated, as all LCOs tested produced root hair deformation in soybean, in a concentration-dependent manner (Table 1). LCOs act at very low (below 10^−7^ M) and very specific concentrations [42,47,48] as is the case for SinCEU-1 and SinCEU-2; however, the LCOs of SinCEU-3 were effective at all concentrations studied in host plant. Furthermore, they were effective in non-host plants, as both height and number of flowers were positively affected with LCOs (Figure 1 and Figure 2). These results are consistent with those obtained by Chen and co-workers [33] with LCOs from *Bradyrhizobium,* suggesting that the receptors responsible for transducing the signal must bind to highly conserved areas of the LCOs, generating a well-defined response in non-legume plants, at least in tomato, which is the plant used in both studies. The mechanisms of action are not yet clarified, although it has been suggested that LCOs might act to control nutrient translocation from source to sink, similarly to phytohormones as IAA (indoleacetic acid) or GA_3_ (gibberellic acid) [33], therefore affecting development.

Some authors argue that UV-B radiation is not an important stress factor compromising plant growth and development, since plants have always been subjected to this type of radiation and have developed mechanisms to survive [49,50,51,52]. However, it is widely accepted that following UV-B radiation exposure, reactive oxygen species (ROS) increase, and ROS scavenging systems are activated to prevent toxic levels [53]. Although ameliorating effects of LCOs in situations of saline stress that involve ROS burst have been reported [38,39], there are no results in the literature on the effect of LCOs on ROS scavenging mechanisms under UV-B radiation stress.

The stress induced by UV-B radiation is evidenced by activation of ROS scavenging mechanisms in UV-controls. ROS behave either as toxic metabolites or as important signaling molecules depending on the concentration [54]. However, when the plant faces biotic and abiotic stress, the production of reactive oxygen species is increased by different mechanisms, and the antioxidant machinery (both enzymatic and non-enzymatic) of the plant is activated to keep ROS below toxic concentrations; our results support this statement as significant increases in most enzymatic activities related to oxidative stress in UV-control as compared to non-stressed control are registered (Figure 6) [1,17,53]. However, the most striking result is the “priming” effect that LCOs induce in tomato plants, which is reported for the first time. LCOs induced a primed state in the plant, which was revealed upon UV-B stress challenge, shown in a faster and more intense response in LCOs-treated plants, by increased enzymatic activities with regards to the UV-B control. This effect is especially outstanding with the LCOs of SinCEU-2 (50), which are able to enhance all activities except SOD.

Our data supports the widely accepted fact that phenolic compounds in general, and flavanols in particular, are molecules with a high antioxidant capacity produced by plants to defend themselves from ROS [17,55], as total phenols and flavanols increase in UV-B controls compared to non-stressed control (Figure 3a,b), behaving as an emergency tool to counteract stress. However, the increase in phenols and flavanols in LCOs-treated plants was not as marked as in UV-controls, and still plants showed significantly higher antioxidant potential (Figure 5), suggesting that LCOs are activating other non-enzymatic antioxidant systems different from those related to phenolic compounds, such as the production of tocopherols, ascorbic acid, glutathione, etc., which ultimately lead to plants having a lower oxidative stress state [26]. Furthermore, this statement supports the priming effect, as alternative protection mechanisms are activated prior to stress challenge.

The high protection against oxidative stress induced by LCOs is shown not only in lower MDA levels (Figure 6) [56,57], but also in photosynthesis, both in Fv/Fm and in Fo parameters (Figure 7). A reduction in Fv/Fm ratio is often an indicator of photoinhibition or other kind of injury to PSII components, especially under stress conditions [58,59,60]. Upon UV-B challenge, the value of Fv/Fm and φPSII significantly decreases, while plants treated with LCOs maintain the values of the non-stressed control. Similarly, an increment on the fluorescence emission of dark-adapted leaves (Fo) indicates photosystem damage [61,62,63]. Plants treated with LCOs have significantly lower Fo values than UV-B controls and even lower than the control, confirming their better physiological state. NPQ values are higher in treatments with LCOs, which again indicates a better physiological state, since it has been shown that an increase in this parameter under stress conditions is a mechanism used by plants to protect PSII [64,65].

The use of elicitors of biological origin or beneficial bacteria with the aim to prevent damage due to unexpected stress situations appears as a very powerful biotechnological tool, contributing to sustainable and respectful agricultural practices [29,31,66,67]. In the case of UV-B radiation, there are some recent studies in which melatonin has been used as an elicitor [68,69], but until now LCOs have never been used in these stressful conditions. This is a first report on the effect of LCOs applied as elicitor to alleviate the oxidative stress effects produced by UV-B. The presented results imply the future deeper approach into the mechanisms triggered by LCOs, to discover the signaling cascades involved in the process, identification of expressed genes, such as certain MAPKs, GST and genes encoding ROS scavenging-related enzymes.

## 4. Materials and Methods

### 4.1. Bacterial Strain Used

The three strains used in this work were isolated in Vietnam from nodules of *Glycine max* [70] and were identified as genus *Ensifer* (formerly *Sinorhizobium*) family Rhizobiaceae. These strains were named by the internal code of our laboratory as: SinCEU-1 (*Ensifer arboris*), SinCEU-2 (*Ensifer fredii*), and SinCEU-3 (*Ensifer fredii*). All strains were grown in TY agar medium [40] containing: 5 gL^–1^ tryptone, 3 gL^−1^ yeast extract, 0.66 g L^−1^ calcium chloride, and 12 g L^−1^ agar.

### 4.2. Isolation and Purification of Lipo-Chitooligosaccharides (LCOs)

Isolation and purification of lipo-chitooligosaccharides from the strains indicated above was done following the protocol described by [71]. Strains were grown in 1 L of yeast mannitol medium (YEM) at 28 °C with constant shaking at 150 rpm for 4 days, when naringenin dissolved in methanol was added to bacterial culture to a final concentration of 5 µM. Ninety-six hours after adding naringenin, bacterial culture was phase-partitioned against 400 mL of 1-butanol (HPLC grade), and then kept overnight in a separatory flask. The upper butanol layer was collected in an evaporation flask and concentrated to 2–3 mL using a rotary evaporator operated at 45 °C. The final extract was re-suspended in 5 mL of 18% acetonitrile and kept in a glass vial at 4 °C.

### 4.3. Degradation of Chitooligosaccharides by Glusulase

Glusulase was prepared from snail juice, mixing 0.5 mL of snail juice (beta-glucuronidase from *Helix pomatia*; SIGMA) with 0.5 mL of 20 mM KCl, and applied to a Biorad biogel P-6 column. Before passing this mixture through the column, it was conditioned with a mix of 10 mM KCl containing 1 mM EDTA at 4 °C. The first 1.5 mL after the void volume was collected and stored at 4 °C. This fraction was used as glusulase. To degrade LCOs, 20 μL of LCOs, 5 μL of 1 M phosphate buffer (pH, 7.1), 25 μL of glusulase, and H_2_O to a final volume of 125 μL were mixed and incubated for 1 h at 37 °C.

### 4.4. Determination of N-Acetyl Glucosamine (GlcNAc) from LCOs

To quantify the amount of N-acetyl glucosamine (GlcNAc) released from LCOs after glusulase reaction, the protocol of [72] was used. Briefly, 125 μL of the GlcNAc released after glusulase reaction was mixed with 25 μL of 0.8 M potassium tetraborate (adjusted to pH, 9.1 with KOH). After incubation at 100 °C for 3 min, samples were placed on ice. After cooling down, 750 μL of a DMAB (p-dimethylaminobenzaldehyde) solution were added. DMAB solution was prepared diluting 10 g in 100 mL of glacial acetic acid, which contained 12.5% of 10 N HCl. This solution is stable for 2 months at 4 °C. Before use, this solution must be diluted with glacial acetic acid (1:9). Subsequently, samples were incubated at 37 °C for 20 min. Samples were cooled on ice, and absorbance at 585 nm was measured against water (care was taken to avoid erratic absorbance readings due to condensation on the cuvettes). As LCOs structures were not known, concentration was estimated by dividing by 4 results of GlcNAc, since the literature reports that LCOs have between 3 and 5 molecules of GlcNAc [73,74,75].

### 4.5. Biological Activity Assay of LCOs

Biological activity of LCOs was confirmed using the root hair deformation assay in soybean as described by [76]. Seeds of soybean cv. OAC Bayfield were surface sterilized in 2.5% sodium hypochlorite for 3 min and washed several times with sterile distilled water. Seeds were germinated on 1% water agar in Petri dishes kept in the dark at 25 °C. After one week lateral roots with ample root hairs were excised and placed on sterile glass slides containing 40–60 µL of each strain LCOs (10^−7^, 10^−8^, 10^−10^, 10^−12^ M) solutions, in triplicate. The slides were then kept in the dark in a sealed moist chamber at 25 °C. Roots were then fixed in a staining solution containing methylene blue (0.02% *w*/*v*) in glycerol (20% *v*/*v*) for 18 h and were observed using light microscopy. Curvature of hairs was recorded in fifty root hairs in each replicate.

### 4.6. Experimental Design to Check the Effect of LCOs on Growth (Height and Flowering) of Tomato Plants

**Figure 8:**
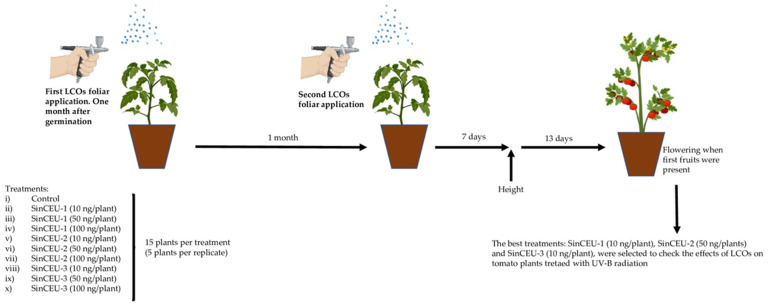
Experimental design to check the effect of LCOs on growth (height and flowering) of tomato plants.

The experimental design is shown in Figure 8. Seeds were sown in 61 cm^3^ germination seedbeds containing coco fiber (Projar Cocopeat). Plants were maintained in a culture chamber (Sanyo MLR350H) with an 18–6 h and 28–25 °C light–dark cycle. One month after germination, plants were transplanted to 500 mL pots containing peat and sand (1:1 v:v). Ten treatments were designed considering LCOs (3) and concentrations (3): (i) control; (ii) plants treated with LCOs from SinCEU-1 (10 ng/plant); (iii) plants treated with LCOs from SinCEU-1 (50 ng/plant); (iv) plants treated with LCOs from SinCEU-1 (100 ng/plant); (v) plants treated with LCOs from SinCEU-2 (10 ng/plant); (vi) plants treated with LCOs from SinCEU-2 (50 ng/plant); (vii) plants treated with LCOs from SinCEU-2 (100 ng/plant); (viii) plants treated with LCOs from SinCEU-3 (10 ng/plant); (ix) plants treated with LCOs from SinCEU-3 (50 ng/plant); (x) plants treated with LCOs from SinCEU-3 (100 ng/plant). Five plants constituted one replicate, and three replicates were used for each treatment. Therefore, fifteen plants constituted each treatment. LCOs were applied by foliar spray 1 month (first inoculation) and 2 months (second inoculation) after transplant. Height was measured 1 week after the second inoculation, and flowering was assessed 20 days after the second inoculation (when first fruits were present).

### 4.7. Experimental Design to Check the Effects of LCOs on Tomato Plants Treated with UV-B Radiation

**Figure 9:**
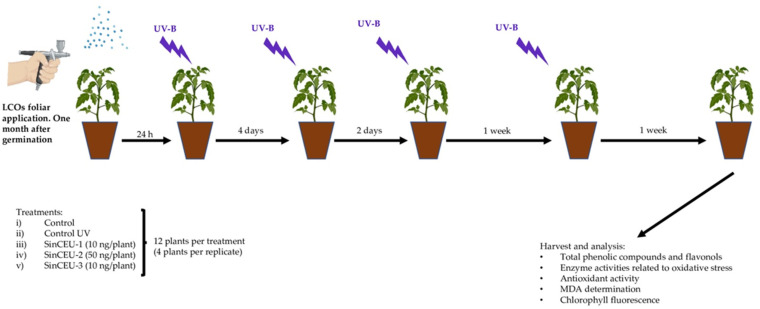
Experimental design to check the effects of LCOs on tomato plants treated with UV-B radiation.

The experimental design is shown in Figure 9. Based on results of the above experiments, the best treatments were selected. LCOs used in this experiment were: SinCEU-1 10 ng/plant (SinCEU-1 (10)), SinCEU-2 50 ng/plant (SinCEU-2 (50)), and SinCEU-3 10 ng/plant (SinCEU-3 (10)). Five treatments were designed: (i) control; (ii) plants irradiated with UV-B (UV-control); (iii) plants treated with LCOs from SinCEU-1 (10 ng/plant) and irradiated with UV-B (SinCEU-1 (10)); (iv) plants treated with LCOs from SinCEU-2 (50 ng/plant) and irradiated with UV-B (SinCEU-2 (50)); and (v) plants treated with LCOs from SinCEU-3 (10 ng/plant) and irradiated with UV-B (SinCEU-3 (10)). Twelve plants constituted one replicate, and three replicates were used for each treatment. Therefore, thirty-six plants constituted each treatment.

Seeds were sown in 61 cm^3^ germination seedbeds, containing peat (Projar PS Seed Pro 5050). Plants were maintained in a culture chamber (Sanyo MLR350H) with an 18–6 h and 28–25 °C light–dark cycle. One month after germination, plants were treated with LCOs. Then, 4 doses of UV-B irradiation supplied by 3 Philips TL 20 W/01 RS lamps, max. 315 nm (3.25 μmol m-2 s-1 photon flux density) for 3 h were delivered after LCOs application. The first one 24 h after LCOs, the second 4 days after, the third 2 days later, and the fourth 1 week later. Seven days after the last irradiation, photosynthesis efficiency was determined, leaves were harvested and powdered in liquid nitrogen. The powder was frozen at −80 °C until analyses. The following parameters were determined: non-enzymatic antioxidants (phenols and flavanols), ROS scavenging enzymes, antioxidant potential, and malondialdehyde (MDA).

### 4.8. Non-Enzymatic Antioxidants (Total Phenolics Compounds and Total Flavonoids)

One gram of leaf powder (Section 2.7) was mixed with 9 mL of 80% methanol. This mix was sonicated for 10 min and centrifuged at 4 °C for 5 min at 3.500 rpm. Supernatant was used to assess total phenolic compounds and flavanols.

Total phenolics compounds (TPC) were determined quantitatively with Folin-Ciocalteau reagent (Sigma Aldrich, St Louis, MO, USA) by colorimetry [77] with modifications, using gallic acid as a standard (Sigma-Aldrich, St. Louis, MO, USA). One mL aliquot of the supernatant was mixed with 250 μL of a 2 N Folin-Ciocalteu reagent (Sigma-Aldrich) and 750 μL of 20% Na_2_CO_3_ solution. After keeping the mix at room temperature for 30 min, absorbance was measured at 760 nm with a UV-Visible spectrophotometer (Biomate 5). A calibration curve was constructed with gallic acid. Results were expressed as mg gallic acid equivalent (GAE) per mg of fresh weight (FW).

Total flavonoids contents were determined quantitatively by the aluminum chloride colorimetric assay [78], using catechin as a standard (Sigma-Aldrich). One mL aliquot of the supernatant solution was added to 10 mL volumetric flask containing 4 mL of distilled water. Three hundred μL of 5% NaNO_2_ were added. Five minutes later 300 μL of 10% AlCl_3_ were added. After 1 min, 2 mL of 1 M NaOH were added, and the total volume was brought to 10 mL with distilled water. The solution was mixed thoroughly, and absorbance was measured against prepared reagent blank at 510 nm. A calibration curve was constructed with catechin. The total flavonoid content of fruit extracts was expressed as mg catechin equivalents (CE) per mg of leaves fresh weight (FW). All samples were analyzed in triplicate.

### 4.9. Enzyme Activities Related to Oxidative Stress

First, extracts for enzyme determinations were prepared by resuspending 10 mg of powder in 1 mL of potassium phosphate buffer 0,1 M pH 7 with 2 mM of PMSF (Phenyl methyl sulfonyl fluoride). This mix was sonicated for 10 min and centrifuged for 10 min at 14.000 rpm. Soluble proteins were measured in the supernatant. All these operations were carried out at 4 °C.

To measure the amount of total protein, 50 µL of supernatant were mixed with 250 µL of Bradford reagent in ELISA 96-well plates. After incubation at room temperature for 5 to 45 min, absorbance at 595 nm was measured in a plate reader (Heales. MB-580). Absorbances were interpolated in a calibration curve constructed from commercial BSA dilutions between 0.05 and 2 mg mL^−1^.

Enzyme activities related to scavenging of ROS were measured spectrophotometrically in the supernatant obtained above. These enzymes activities were: catalase (CAT, EC 1.11.1.6), ascorbate peroxidase (APX, EC 1.11.1.11), superoxide dismutase (SOD, EC 1.15.1.1), guaiacol peroxidase (GPX, EC 1.11.1.7), dehydroascorbate reductase (DHAR; EC 1.8.5.1), glutathione reductase (GR, EC 1.6.4.2) and monodehydroascorbate reductase (MDHAR; EC 1.6.5.4). All were expressed as μmol/mg protein and min, except SOD that was expressed as % inhibition/mg protein.

SOD activity was determined following the method proposed by the SOD activity detection kit from Sigma-Aldrich (SOD Assay Kit-WST; Dojindo EU). This method is based on the assumption that the rate of the reduction with O_2_ is linearly related to the xanthine oxidase (XO) activity and inhibited by SOD. Inhibition activity of SOD can be determined by colorimetric method.

CAT was measured following the method of Garcia–Limones and co-workers [79]. Nine hundred and eighty μL of 50 mM potassium phosphate buffer (pH 7.0) were mixed with 100 μL of enzyme extract. The reaction was started by adding 120 μL of H_2_O_2_ 200 mM. Immediately, the decrease in A240 produced by H_2_O_2_ breakdown was recorded. An extinction coefficient of 36 mM^−1^ cm^−1^ was used to calculate activity.

APX was measured following the method of Garcia–Limones and co-workers [79]. Eight hundred and sixty μL of 50 mM potassium phosphate buffer pH 7.0 were mixed with 120 μL of sodium ascorbate 2.5 mM and 100 μL of enzyme extract. The reaction was started by adding 120 μL of H_2_O_2_ 50 mM. The oxidation of ascorbate was determined by the decrease in absorbance at 290 nm. An extinction coefficient of 2.8 mM^−1^ cm^−1^ was used to calculate activity.

GPX was measured following the method of Garcia–Limones and co-workers [79]. Eight hundred eighty μL of 100 mM potassium phosphate buffer (pH 6.5) were mixed with 120 μL of guaiacol 150 mM and 200 μL of enzyme extract. Adding 1 μL of H_2_O_2_ started the reaction and the oxidation of guaiacol was determined by the increase in absorbance at 470 nm. An extinction coefficient of 26.6 mM^−1^ cm^−1^ was used to calculate activity.

MDHAR activity was measured following the method of Xu and co-workers [80]. Eight hundred and sixty μL of 50 mM potassium phosphate buffer (pH 7.6) were mixed with 120 μL of NADH 2 mM and 120 μL of ascorbic acid (AsA) 25 mM. Then, 1 unit of ascorbate oxidase was added, and the tube was incubated 20 min at 35 °C. Adding 100 μL enzyme extract started the reaction and the reduction of monodehydro ascorbate was determined by the decrease in A340. An extinction coefficient of 6.22 mM^−1^ cm^−1^ was used to calculate activity.

DHAR activity was measured following the method of Xu and co-workers [80]. Seven hundred forty μL of potassium phosphate buffer 50 mM (pH 7.0) were mixed with 120 μL of reduced glutathione 25 mM, 120 μL of Dehydroascorbate 2 mM and 120 μL of EDTA 1mM. Adding 100 μL of enzyme extract started the reaction and the reduction of dehrydroascorbate was determined by the decrease in A265. An extinction coefficient of 14 mM^−1^ cm^−1^ was used to calculate activity.

GR was measured following the method of Garcia–Limones and co-workers [79]. Seven hundred forty μL of 50 mM potassium phosphate buffer pH 7.5, were mixed with 120 μL of DNTB 10 mM, 120 μL of NADPH 1 mM, 120 μL of oxidized glutathione 10 mM, and 180 μL of enzyme extract in a final volume of 1.2 mL. Adding 100 μL of enzyme extract started the reaction oxidation of NADH was determined by the increase in A340. An extinction coefficient of 6.2 mM^−1^ cm^−1^ was used to calculate activity.

In all cases, all components of the reaction except the enzyme extract, which was replaced by buffer, were the blanks of each assay. In the case of the GR assay, an additional blank without oxidized glutathione was included to account for the presence in the extracts of other enzyme activities able to oxidize NADPH.

### 4.10. Determination of Antioxidant Activity with the β-Carotene-Linoleic Acid Bleaching Method

Determination of antioxidant activity using a β-carotene/linoleic acid system was carried out according to the method described by Matthäus [81]. This method is based on the following reaction: the free linoleic acid radical formed upon the abstraction of a hydrogen atom from one of its methylene groups attacks the β-carotene molecules, which lose their double bonds and, therefore, their characteristic orange color. The bleaching rate of the β-carotene solution was determined by the difference between the initial reading in spectral absorbance at 470 nm at time 0 min and after 60 min.

Briefly, 40 mg of linoleic acid and 400 mg of Tween 20 were placed into a flask, and 1 mL of a solution of β-carotene (3.34 mg mL^-1^) in chloroform was added. Chloroform was removed by rotary evaporation at 40 ºC. Then 100 mL of distilled water was added slowly to the residue and the solution was vigorously agitated to form a stable emulsion. Five mL of this emulsion were mixed with 0.2 mL of supernatant, and the absorbance was measured at 470 nm, immediately (t0), against a blank consisting of the emulsion without β-carotene. Tubes were placed in a water bath at 40 °C, and the absorbance was measured every 15 min up to 60 min. Results are expressed as % of inhibition = 1 − [(Abst0_sample_ − Abs60 min_sample_)/(Abst0_control_ − Abs60 min_control_)] × 100.

### 4.11. Malondialdehyde (MDA) Determination

Malondialdehyde (MDA) content was evaluated following the method of Hu and co-workers [82]. Two hundred twenty-five mg of powder were mixed with 2 mL of reaction solution containing 20% (*v*/*v*) trichloroacetic acid (TCA) and 0.5% (*v*/*v*) thiobarbituric acid (TBA). After heating at 95 °C for 30 min and cooling to room temperature, the mixture was centrifuged at 6030 g for 20 min. Absorbance of the supernatant was determined spectrophotometrically at 532 and 600 nm. The concentration of MDA was calculated using the formula MDA (nmol g FW^−1^) = [(OD_532_ − OD_600_)]/(ε × FW), where FW is fresh weight and ε the extinction coefficient (155 mM^−1^ cm^−1^). Data were expressed as μmol g FW^−1^ (fresh weight).

### 4.12. Chlorophyll Fluorescence Measurements

Chlorophyll fluorescence was measured with a pulse amplitude modulated (PAM) fluorometer (HansatechFM2, Hansatech, Inc., Norfolk, UK) on 1 h dark-adapted leaves. The minimal fluorescence (Fo; dark adapted minimum fluorescence) was measured with weak modulated irradiation (1 molm^−2^s^−1^). Maximum fluorescence (Fm) was determined for the dark-adapted state by applying a 700 ms saturating flash (9000 molm^−2^s^−1^). The variable fluorescence (Fv) was calculated as the difference between the maximum fluorescence (Fm) and the minimum fluorescence (Fo). The maximum photosynthetic efficiency of photosystem II (maximal PSII quantum yield) was calculated as Fv/Fm. Immediately, the leaf was continuously irradiated with red–blue actinic beams (80 molm^−2^s^−1^) and equilibrated for 15 s to record Fs (steady-state fluorescence signal). Following this, another saturation flash (9000 molm^−2^s^−1^) was applied and then Fm’ (maximum fluorescence under light adapted conditions) was determined. Other fluorescent parameters were calculated as fol-lows: the effective PSII quantum yield φPSII = (Fm’ − Fs)/Fm’ and non-photochemical quenching coefficient NPQ = (Fm − Fm’)/Fm’.

### 4.13. Statistical Analysis

To determine the statistical differences between the results obtained, *t*-test and analysis of variance (ANOVA) were used. For plant height and number of flowers, each treatment was compared to control by *t*-test. One-way ANOVA was performed in the rest of parameters, where all treatments were compared among them; when significant differences appeared, (*p* < 0.05), a Fisher test was used [83].

In both cases, prior to analysis homoscedasticity and normality of the variance was checked, meeting requirements for analysis. Analyses were performed with Statgraphics plus 5.1 for Windows.

## 5. Conclusions

Taking into account the above, the results of this work demonstrate the beneficial effects of LCOs, both under normal conditions and under stress conditions due to UV-B radiation. Under stress conditions, LCOs improve the physiological status of plants, lowering oxidative stress as indicated by MDA. Effects are evidenced in the improvement of photosynthetic parameters, one of the first physiological processes affected by the presence of high levels of ROS. LCOs improve ROS scavenging systems, modifying the functioning of the ascorbate–glutathione cycle. Therefore, although more studies and the deepening of certain aspects are necessary, LCOs could be used as elicitors in agricultural systems subjected to UV-B stress.

## Figures and Tables

**Table 1:** Percentage of deformed root hairs.

	Concentration of LCOs (M)				
LCOs from:	1.00 × 10^−7^	1.00 × 10^−8^	1.00 × 10^−10^	1.00 × 10^−12^	without LCOs
Control	-	-	-	-	12%
SinCEU-1	18.57%	26.92% *	21.59%	11.33%	-
SinCEU-2	14.44%	41.05% *	17.33%	24.29%	-
SinCEU-3	34% *	34.15% *	34.15% *	32.4% *	-

* indicate significant differences with control (*p* < 0.05).

## Data Availability

The data presented in this study are available in the article.
